# A mouse model of Zika virus sexual transmission reveals a limited transmission window shorter than viral RNA persistence in semen

**DOI:** 10.1371/journal.ppat.1014420

**Published:** 2026-07-14

**Authors:** Jia-Tong Chang, Zhan-Zhan Bian, Lin-Shen-Yang Liu, Dong-Ying Fan, Xiao-Feng Qin, Nan Zhang, Hui Chen, Wei Yang, Pei-Gang Wang, Jing An

**Affiliations:** 1 Department of Microbiology, School of Basic Medical Sciences, Capital Medical University, Beijing, China; 2 Laboratory for Clinical Medicine, Capital Medical University, Beijing, China; 3 Department of Immunology, School of Basic Medical Sciences, Capital Medical University, Beijing, China; 4 Institute of Laboratory Animal Science, Chinese Academy of Medical Sciences Institute of Laboratory Animal Sciences, Beijing, China; University of California Davis, UNITED STATES OF AMERICA

## Abstract

Sexual transmission of Zika virus (ZIKV) presents a distinct public health challenge, yet its kinetics and mechanisms remain poorly defined due to the lack of suitable immunocompetent animal models. Here, we established a mouse model of ZIKV sexual transmission using human STAT2 knock-in (hSTAT2 KI) mice. We show that infected males efficiently transmit ZIKV to females via mating. Beyond sperm, non-sperm components in semen substantially contributed to transmission. ZIKV sexual transmission was time-dependent, peaking early at 5 days post-infection (dpi) and declining rapidly thereafter, with transmission virtually ceasing by 40 dpi—a short transmission window that did not align with the prolonged presence of ZIKV RNA in sperm, suggesting clearance of infectious virus or rising antibody responses. Both African and Asian ZIKV strains displayed similar transmission capacities, indicating evolutionary conservation of this trait. In females, infection resulted in viremia and viral RNA detection in multiple organs of the reproductive tract, with predominant localization in the vagina, where it triggered localized lipid metabolic disturbances with minimal impact on upper tract tissues. Our model recapitulates key aspects of human ZIKV sexual transmission, offering a robust platform for mechanistic study and intervention development.

## Author summary

Zika virus (ZIKV) is primarily transmitted by mosquitoes but can also spread sexually, especially from men to women. Although ZIKV RNA can persist in semen for months, the duration of actual sexual transmissibility remains unclear. Here, we established an immunocompetent mouse model of ZIKV sexual transmission using human STAT2 knock-in (hSTAT2 KI) mice. Infected males efficiently transmitted ZIKV to females during mating, with transmission peaking at 5 days post-infection, declining by 20 days, and disappearing by 40 days, despite persistent viral RNA in sperm. These findings suggest that RNA persistence does not necessarily indicate continued infectiousness. We also found that non-sperm components of semen contributed substantially to transmission, because males remained partially infectious even after epididymal ligation blocked sperm release. In females, infection was mainly restricted to the vagina, where it caused limited tissue damage but altered lipid metabolism-related gene expression. Together, these findings provide new insight into the kinetics and mechanisms of ZIKV sexual transmission and establish a valuable experimental platform for future studies of prevention and intervention strategies.

## Introduction

Zika virus (ZIKV), a member of the genus *Orthoflavivirus* within the family Flaviviridae, has emerged as a global health threat, being linked to severe disorders such as microcephaly and Guillain-Barré syndrome [1]. While primarily transmitted through the bite of *Aedes* mosquitoes, outbreaks in the last decade have confirmed that ZIKV can also be spread sexually—predominantly from males to females—posing significant challenges for public health control and reproductive health [2,3].

Clinical observations indicate that most sexual transmission events occur within 19 days after the onset of symptoms [4]. However, sporadic cases have been reported in which sexual transmission occurred between 34 and 41 days after symptom onset [5]. Consequently, the definitive duration and overall contribution of sexual transmission to ZIKV spread remain uncertain. Sexual transmission of ZIKV relies on viral infection of the male reproductive tract. Infected male patients have been reported to exhibit symptoms such as oligospermia [6] and hematospermia [7]. Notably, infectious ZIKV particles have been detected in semen for up to 69 days, while viral RNA can persist for as long as 188 days [8]. However, clinical data are often limited by incomplete information and inter-individual variability, hindering systematic investigation of ZIKV sexual transmission kinetics and mechanisms. This gap in knowledge impedes a deeper understanding of viral transmission dynamics and the development of effective control strategies.

Therefore, establishing a reliable small-animal model to study ZIKV sexual transmission, evaluate its kinetics, and elucidate its mechanisms is of critical importance. Immunodeficient mice, which lack all or part of the interferon response, are more susceptible to ZIKV infection than wild-type mice and have thus been widely used in ZIKV research. The AG129 mice (lacking IFNAR/IFNGR) [9,10] and *Ifnar1*^−/−^ mice [11] have been employed to study ZIKV sexual transmission and have provided valuable insights. However, these models often develop severe pathological manifestations or even succumb to infection [12,13]. Their altered immune responses fail to accurately reflect the immune status and disease progression observed in humans, limiting their utility for modeling sexual transmission and investigating prolonged viral shedding. In addition, the use of wild-type mice treated with an anti-IFNAR1 blocking antibody is also constrained by high costs and inconsistent efficacy [14].

Although ZIKV can infect wild-type C57BL/6J mice via intravaginal inoculation [15], this approach does not account for the mating behavior involved in sexual transmission. Intravaginal infection bypasses key factors involved in natural transmission, including viral accumulation in semen, the complex cellular microenvironment of the male reproductive tract, and the critical interactions between semen and the female reproductive tract. In addition to wild-type mice, researchers have developed an immunocompetent model by introducing human STAT2 into the mouse Stat2 locus (hSTAT2 KI), which enables ZIKV to effectively antagonize the murine innate immune response and establish infection [16]. However, hSTAT2 KI mice used to study ZIKV transmission have only been infected via intravaginal inoculation [17].

In this study, we established a model of ZIKV sexual transmission using hSTAT2 KI mice and evaluated the transmission kinetics of different ZIKV strains and the time-dependent dynamics. Although ZIKV RNA persisted in the male reproductive tract, the window of sexual transmissibility is relatively short. Both African and Asian ZIKV lineages exhibited comparable sexual transmission efficiency. Moreover, in immunocompetent hosts, the impact of sexually transmitted ZIKV on the non-pregnant female reproductive tract was primarily confined to the vagina, where the infection triggered localized lipid metabolic disturbances without inducing significant cell death or broad immune activation. Overall, the pathological impact during the acute phase in non-pregnant females appears limited. These findings reveal the dynamics and mechanistic determinants of ZIKV sexual transmission and identify the vagina as the primary target of infection, along with its associated pathological alterations. Our study provides a critical experimental foundation for assessing the risks of ZIKV sexual transmission, elucidating its mechanisms, and developing targeted prevention and control strategies.

## Results

### Sexual transmission of ZIKV in hSTAT2 KI mice

**Fig 1:**
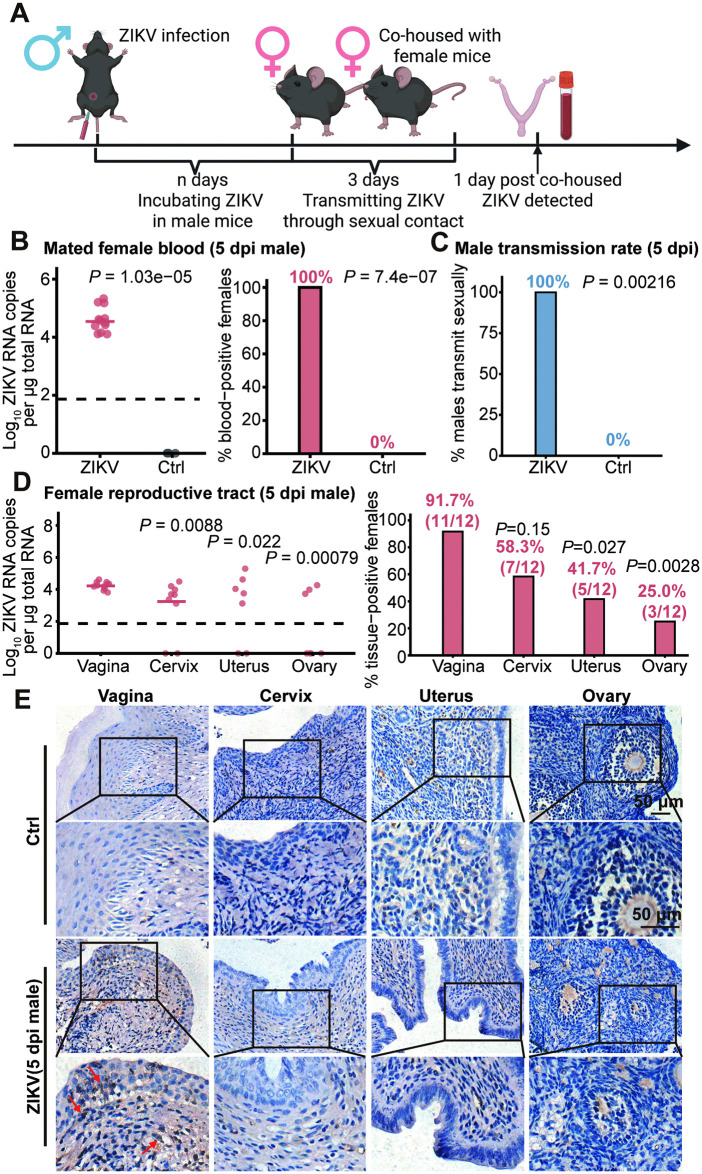
Sexual transmission of ZIKV from male to female hSTAT2 KI mice. **(A)** Experimental design. Male hSTAT2 KI mice were intraperitoneally inoculated with 1 × 10⁴ PFU of ZIKV (SMGC-1 strain) and mated with healthy females at a 1:2 ratio for three days. Samples (blood and reproductive tissues) were collected from females one day after mating for viral detection. This figure was created in BioRender. Chang, J. (2026) and is published under a CC BY 4.0 license. https://BioRender.com/btltqf0. BioRender.com. **(B)** Viral RNA loads in female blood and infection rate. Female mice mated with males at 5 days post-infection (dpi) were analyzed. Left: viral RNA loads in blood. Right: infection rate (n = 12). **(C)** Proportion of infected males at 5 dpi that successfully transmitted ZIKV to females. A male was considered a transmitter if at least one of the co-housed females developed detectable viremia following mating (n = 6). **(D)** Viral RNA loads and detection rates in female reproductive organs. Left: viral RNA loads per organ. Right: ZIKV detection rates (n = 6). **(E)** Immunohistochemical localization of ZIKV E protein in female reproductive tissues. (Scale bar, 50 μm). Red arrows indicate positive signals.

To determine whether ZIKV can be transmitted via mating behavior in hSTAT2 KI mice, 6–8-week-old males were intraperitoneally inoculated with 1 × 10⁴ PFU of ZIKV. At defined days post-infection (dpi), each male was mated with two uninfected adult females for three days. A control group of infected males co-housed with uninfected males under the same conditions was included to exclude the possibility of non-sexual transmission. One day after co-housing, viremia was assessed in both mated female and male mice, and viral loads were quantified in the vagina, cervix, uterus, and ovary of female mice (Fig 1A).

Viremia was used to detect infection in mated mice. Reverse transcriptase quantitative polymerase chain reaction (RT-qPCR) revealed that after mating with males infected at 5 dpi, all female mice (12/12) had detectable ZIKV RNA in blood with similar levels, whereas no viremia was detected in control males (Fig 1B). A male was considered a transmitter if at least one of the co-housed females developed detectable viremia following mating. Thereby, the male-to-female transmission rate was 100% (Fig 1C).

Viral RNA detection in female reproductive tissues showed the highest detection frequency in the vagina (11/12), followed by the cervix (7/12), uterus (5/12), and ovary (3/12) (Fig 1D). The detection rate declined along the anatomical axis of the reproductive tract, suggesting a bottom-up ascending pattern of viral spread. Immunohistochemical staining for the ZIKV envelope (E) protein confirmed that viral antigens were predominantly localized in the vaginal epithelial layer and the underlying lamina propria (Fig 1E), consistent with RT-qPCR results.

To further confirm the presence of infectious virus in co-housed females, plaque assays were performed using vaginal and blood homogenate supernatants from mated females. Consistent with the RT-qPCR results, distinct viral plaques were observed in samples from the ZIKV group, whereas no plaques were detected in the control group (S1A Fig). These results demonstrate that ZIKV is efficiently transmitted from males to females through mating, with the vagina serving as the primary site of viral entry and replication.

Viral loads were compared using the Wilcoxon rank-sum test, and infection rates were compared using Fisher’s exact test.

### Transcriptional signatures in the female reproductive system following sexual transmission

To evaluate the physiological and molecular impact of sexually transmitted ZIKV on the female reproductive system, female mice mated with either uninfected males (control) or ZIKV-infected males (infection group, 5 dpi) were monitored daily. No significant differences in body weight were observed between the two groups (S1B Fig). However, viral RNA was detected in the blood of all females in the infection group (S1C Fig), confirming successful infection.

**Fig 2:**
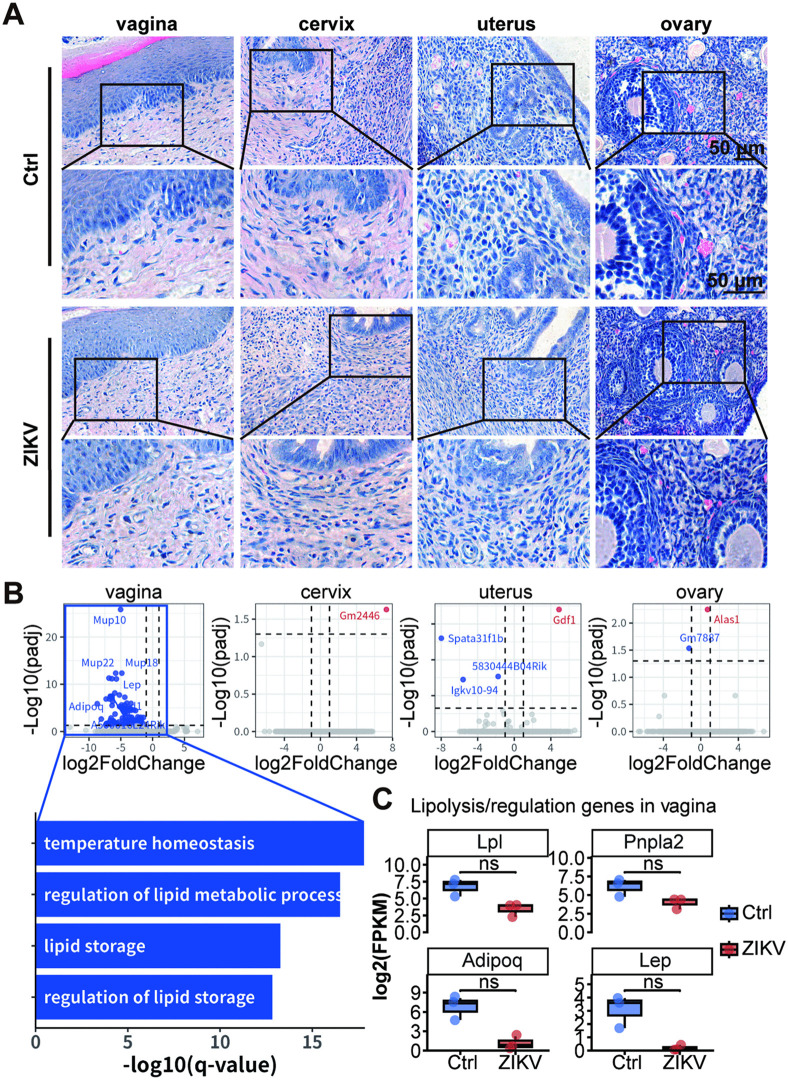
Pathological and transcriptional signatures in the female reproductive tract following sexual transmission of ZIKV. **(A)** Representative H&E-stained sections of the vagina, cervix, uterus, and ovary from female mice (n = 3). (Scale bar, 50 μm). **(B)** Analysis of differentially expressed genes (DEGs) and Gene Ontology (GO) enrichment. DEGs were identified by comparing ZIKV group females (mated with infected males) with control group females (mated with uninfected males) using DESeq2 with criteria of |log₂ fold change| > 1 and adjusted p-value < 0.05. GO enrichment analysis was performed using the clusterProfiler package. **(C)** Expression levels of key genes related to lipolysis and endocrine regulation in vaginal tissue. Data are presented as mean ± SEM (n = 3 per group). Statistical analysis was performed using the Mann-Whitney U test.

Hematoxylin and eosin (H&E) staining was performed to assess histopathological changes in the reproductive tract (Fig 2A). No significant tissue injury or histopathological abnormalities were observed in the vagina, cervix, uterus, or ovary of infected females compared to uninfected controls.

To further investigate molecular alterations, bulk RNA sequencing was performed on vaginal, cervical, uterine, and ovarian tissues from mated hSTAT2 KI females (ZIKV group females mated with infected males versus control group females mated with uninfected males). Differential expression analysis revealed that the vagina exhibited the highest number of differentially expressed genes (DEGs), most of which were downregulated (Fig 2B). Among these, genes of the major urinary protein (Mup) family—implicated in metabolic regulation and immune responses - were notably suppressed. Gene Ontology (GO) enrichment analysis showed significant downregulation of pathways associated with thermoregulation, regulation of lipid metabolic processes, and lipid storage in vaginal tissues of infected mice. In particular, genes involved in lipid catabolism and energy balance, including *Lpl*, *Pnpla2*, *Adipoq*, and *Lep*, displayed a decreasing trend (Fig 2C). In contrast, genes associated with cholesterol and fatty acid metabolism showed no significant alterations (S2A Fig). Collectively, ZIKV induces localized dysregulation of lipid metabolism in the vagina under the conditions examined. To assess tissue injury and immune onset, we examined the transcriptional profiles of genes associated with cell death and interferon responses in vaginal tissues. No significant changes were observed in either pathway (Figs 2C, S2B).

Furthermore, no significant transcriptional disparities were observed in the cervix, uterus, or ovary compared with controls (Fig 2B). These data indicate that, at the acute time point analyzed (one day after mating with 5 dpi males), the most pronounced transcriptional changes were confined to the vagina, with minimal alterations detected in the upper reproductive tract. However, whether these acute-phase changes have any longer-term implications for reproductive health remains unknown, and further studies with larger sample sizes and extended observation periods are needed.

### Distribution of ZIKV in the male reproductive tract of hSTAT2 KI mice

To investigate the mechanism of sexual transmission, male hSTAT2 KI mice infected with ZIKV were monitored daily. Consistent with our previous findings [18], infected males exhibited only transient body weight loss on the first and second day post-infection, after which body weight returned to baseline levels (S3A Fig). All infected mice survived for the duration of the experiment (S3B Fig).

H&E staining of the male reproductive system was performed at 5 dpi to assess early histopathological changes following ZIKV infection (S3C Fig). No significant histopathological abnormalities were observed in the testis, epididymis, prostate, or seminal vesicle, and the overall tissue architecture appeared comparable to that of uninfected controls. The seminiferous tubules retained normal structural organization, and no convincing evidence of inflammatory cell infiltration, hemorrhage, or vascular congestion specifically attributable to ZIKV infection was identified at this early time point.

Collectively, these findings indicate that during the early acute phase of infection (5 dpi), ZIKV infection in hSTAT2 KI mice is associated with minimal histopathological alterations in the male reproductive organs, with overall tissue architecture largely preserved.

**Fig 3:**
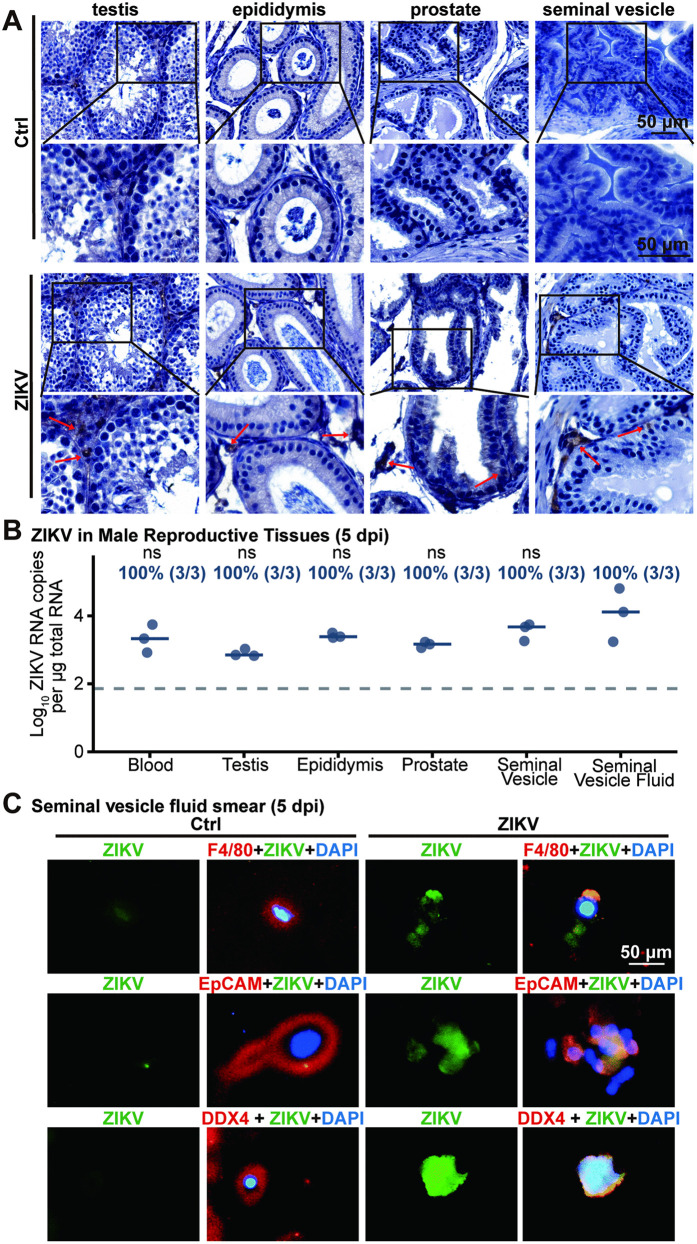
Viral detection in male reproductive organs of ZIKV-infected hSTAT2 KI mice at 5 dpi. **(A)** Representative immunohistochemical staining for the ZIKV E protein in the testes, epididymides, prostate, and seminal vesicles. Positive staining (brown) indicates the presence of ZIKV E protein. Sections were counterstained with hematoxylin. Red arrows indicate representative positive signals. (Scale bar, 50 μm). **(B)** Viral RNA loads in blood and reproductive tissues (testes, epididymis, prostate, seminal vesicles, and seminal vesicle fluid) (n = 3). Data are presented as individual data points with median (horizontal bar). Mann-Whitney U test was used for statistical analysis. **(C)** Immunofluorescence staining of seminal vesicle fluid smears. ZIKV E protein was detected in DDX4⁺ germ cells, EpCAM⁺ epithelial cells, and F4/80⁺ macrophages. Scale bar, 50 μm.

Although ZIKV infection did not induce severe histopathological damage, immunohistochemical staining detected ZIKV E protein in multiple regions of the male reproductive tract. In the testes, ZIKV-positive signals were primarily localized to the interstitial compartment, where potential cell types include Leydig cells, resident macrophages, or infiltrating immune cells. In the epididymis, positive staining was mainly observed in interstitial areas surrounding the epididymal ducts, while in the seminal vesicles, ZIKV antigen was detected within interstitial regions of the glandular tissue. In the prostate, most ZIKV-positive signals were also localized to the interstitium, although occasional epithelial cells lining the glandular structures exhibited positive staining (Fig 3A). Overall, these observations suggest that ZIKV infection in the male reproductive system is predominantly associated with interstitial cell populations, although the precise cellular identity cannot be definitively determined based on morphology alone. Consistent with these observations, ZIKV RNA was detected in both the blood and reproductive organs of infected male hSTAT2 KI mice (Fig 3B). Notably, seminal vesicle fluid displayed slightly higher viral RNA load than other reproductive tissues and blood.

To further confirm the presence of infectious virus, plaque assays were performed using blood and seminal vesicle fluid homogenate supernatants from infected males. Consistent with the RT-qPCR results, distinct viral plaques were observed in samples from the ZIKV-infected group, whereas no plaques were detected in the control group (S3D Fig).

To determine which cell types were associated with viral signals in the seminal vesicle fluid, we performed immunofluorescence staining of seminal vesicle fluid smears. ZIKV E protein signals were detected in cells positive for markers of spermatogenic cells (DDX4), epithelial cells (EpCAM), and macrophages (F4/80). In each panel, the rightmost images represent merged channels (DAPI, ZIKV E protein, and the indicated cellular marker), demonstrating co-localization of viral antigen with these cell-type markers (Fig 3C). These results suggest that multiple cell populations present in seminal vesicle fluid may harbor ZIKV. Thus, several components of the male reproductive system appear susceptible to ZIKV infection and may serve as potential reservoirs contributing to sexual transmission.

### Sexual transmission of ZIKV in epididymal-ligated hSTAT2 KI mice

To define the role of non-sperm components in semen for ZIKV sexual transmission, bilateral epididymal ligation was performed on adult male hSTAT2 KI mice. This surgical procedure blocks the release of testicular and epididymal contents, including sperm and epididymal secretions, thereby allowing assessment of ZIKV sexual transmission efficiency in their absence.

**Fig 4:**
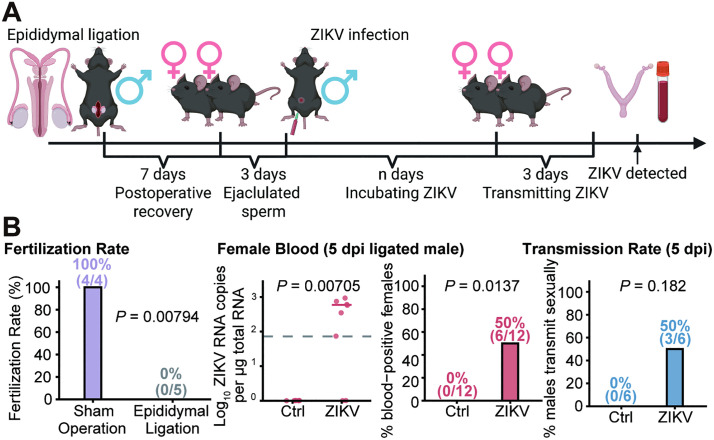
Sexual transmission of ZIKV by epididymal-ligated hSTAT2 KI males. **(A)** Experimental design. Adult males underwent bilateral epididymal ligation, recovery, and sequential mating before and after ZIKV infection. This figure was created in BioRender. Chang, J. (2026) and is published under a CC BY 4.0 license. https://BioRender.com/jk49hoj. **(B)** Pregnancy rate of females mated with sham-operated or epididymal-ligated males (n = 4-5). Statistical analysis was performed using Fisher’s exact test. **(C)** Viral RNA loads in the blood of females mated with infected epididymal-ligated males at 5 dpi. ZIKV RNA was quantified by probe-based RT-qPCR. Statistical analysis was performed using the Mann-Whitney U test. **(D)** Infection rate of ZIKV in mated females (n = 12). Data are presented as percentages. Statistical analysis was performed using Fisher’s exact test. **(E)** Proportion of infected epididymal-ligated males that successfully transmitted ZIKV to females with different ZIKV strains. A male was considered a transmitter if at least one of the co-housed females developed detectable viremia following mating (n = 6 males per group). Statistical analysis was performed using Fisher’s exact test.

As outlined in the experimental design (Fig 4A), adult male hSTAT2 KI mice underwent bilateral epididymal ligation and were allowed to recover for seven days. The first mating was performed to deplete pre-existing sperm stored in the seminal vesicles. Subsequently, the males were intraperitoneally inoculated with ZIKV, and a second mating was conducted following the protocol mentioned before.

To confirm the success of the surgical model, a sham-operated group and a ligated group were included. A second prolonged mating was performed five days after the first mating to assess fertility. The sham group achieved a 100% pregnancy rate, whereas no pregnancies occurred in the ligated group (Fig 4B), demonstrating effective blockage of sperm transport.

Remarkably, ZIKV was detectable in mated females with ligated males infected at 5 dpi (Fig 4C), with an infection rate of 50% (Fig 4D). The sexual transmission efficiency remained as high as 50% even in the absence of sperm (Fig 4E). These results indicate that even after surgical ligation of sperm transport pathways, male mice retain potential for sexual transmission of ZIKV, suggesting that vasectomized human males may still pose a risk of transmitting the virus sexually.

### Similar sexual transmissibility between different ZIKV strains

**Fig 5:**
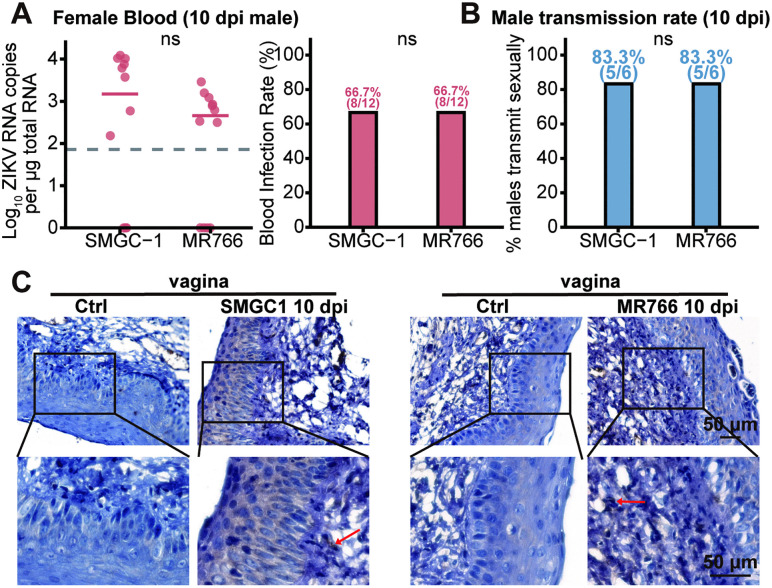
Similar sexual transmissibility between African and Asian ZIKV strains in hSTAT2 KI male mice. **(A)** ZIKV RNA loads in blood and infection rate in mated females. Left: viral RNA loads in blood at 10 dpi. Statistical analysis was performed using the Mann-Whitney U test. Right: infection rate (n = 12). Statistical analysis was performed using Fisher’s exact test. **(B)** Proportion of infected males successfully transmitted ZIKV to females with different ZIKV strains. A male was considered a transmitter if at least one of the co-housed females developed detectable viremia following mating (n = 6 males). Data are presented as percentages. Statistical analysis was performed using Fisher’s exact test. **(C)** Immunohistochemical staining of female vagina following infection ZIKV. Left: Asian strain (SMGC-1). Right: African strain (MR766). Positive signals (brown) indicate the presence of ZIKV E protein. Sections were counterstained with hematoxylin. Red arrows indicate representative positive signals (scale bar, 50 μm).

Previous studies have identified several key mutations in ZIKV that enhanced its mosquito transmissibility and neurovirulence, contributing to the 2015 outbreak and global spread [19]. To evaluate potential differences in sexual transmissibility among ZIKV strains, hSTAT2 KI male mice were infected with either the Asian lineage strain (SMGC-1) or the ancestral African lineage strain (MR766). Because transmission rates at 5 dpi were uniformly high across strains (100%, Fig 1C), potentially masking strain-specific differences, mating experiments were performed at 10 dpi to broaden the temporal window and increase the discriminatory power of the model. Notably, both ZIKV strains successfully established sexual transmission, as evidenced by detectable viremia in mated females, with no significant difference in infection rates between the two groups (Fig 5A). Consistently, no statistically significant difference was observed in sexual transmissibility between males infected with the Asian or African strains (Fig 5B).

Histological examination and immunohistochemical staining for the ZIKV E protein revealed comparable tissue pathology and viral antigen localization between the two strains. ZIKV was predominantly localized within the vaginal epithelium and mucosal layer (Figs 5C and S4), consistent with our earlier findings (Fig 1E). Despite their phylogenetic divergence, both ZIKV strains exhibited similar sexual transmissibility under our experimental conditions. These findings suggest that the capacity for sexual transmission may be shared by both African and Asian lineage ZIKV strains, although confirmation using additional strains and animal models will be necessary to determine whether this trait is evolutionarily conserved.

### Time-dependent decline of ZIKV sexual transmissibility

Although prolonged RNA shedding of ZIKV has been observed in human semen, even up to 188 days [8], the window of sexual transmissibility remains to be determined. We therefore systematically evaluated and compared the kinetics of ZIKV shedding and sexual transmission in male hSTAT2 KI mice.

**Fig 6:**
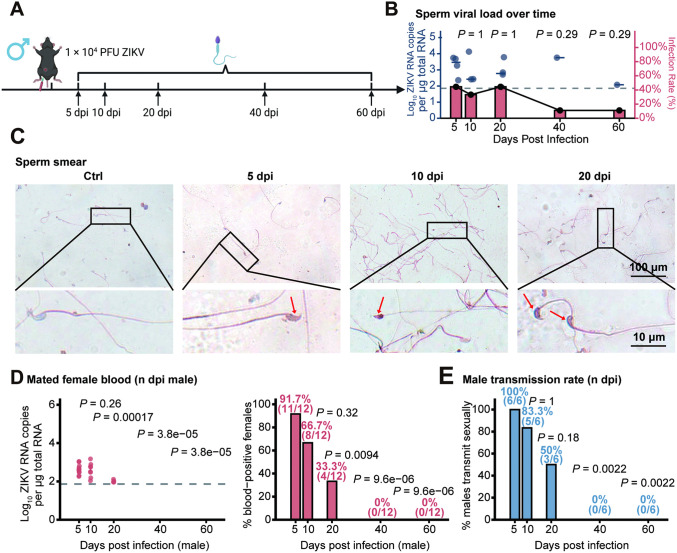
Time-dependent decline in ZIKV sexual transmissibility in hSTAT2 KI male mice. **(A)** Experimental design. Schematic of sample collection from male reproductive systems at 5, 10, 20, 40, and 60 dpi. This figure was created in BioRender. Chang, J. (2026) and is published under a CC BY 4.0 license. https://BioRender.com/wuqon2j. **(B)** Viral RNA levels (blue scatter) and detection rates (red bars) in sperm determined by RT-qPCR (n = 9). Fisher’s exact test was used to compare the infection rate at each time point versus that at 5 dpi. **(C)** Immunohistochemical staining of ZIKV E protein combined with Papanicolaou staining of sperm smears at indicated time points. ZIKV antigen (brown, indicated by red arrows) was detected by IHC prior to Papanicolaou counterstaining. Papanicolaou staining differentially colors sperm components: nuclei appear blue-purple (hematoxylin), acrosomes orange-pink (Orange G6), and cytoplasmic/tail structures blue-green (EA50). **(D)** ZIKV RNA loads in blood and infection rates in females mated with males infected at various dpi. Left: viral RNA loads in blood. Statistical analysis was performed using the Mann-Whitney U test comparing each time point to 5 dpi. Right: infection rates (n = 12 females). Statistical analysis was performed using Fisher’s exact test comparing each time point to 5 dpi. **(E)** Proportion of infected males successfully transmitted ZIKV to females at various dpi. A male was considered a transmitter if at least one of the co-housed females developed detectable viremia following mating (n = 6 males). Statistical analysis was performed using Fisher’s exact test comparing each time point to 5 dpi.

ZIKV RNA was detected in sperm isolated from male hSTAT2 KI mice at various time points post-infection (Fig 6A). The detection rate in sperm remained relatively stable during the first 20 days, ranging from 33.3% to 44.4% at 5, 10, and 20 dpi (Fig 6B). Sperm smears were subjected to immunohistochemical staining for the viral E protein, combined with Papanicolaou staining to assess sperm morphology. As shown in Fig 6C, ZIKV-positive signals (brown, indicated by red arrows) were observed in sperm collected from the epididymis at 5, 10, and 20 dpi, with viral antigen predominantly localized to the sperm head. The brown IHC signal was clearly distinguishable from the blue-purple nuclear staining, orange-pink acrosome staining, and blue-green cytoplasmic/tail staining produced by the Papanicolaou method. Notably, the detection rate of ZIKV-positive sperm (44.4%) was lower than the sexual transmission rate observed at 5 dpi (100%), supporting the contribution of non-sperm components (e.g., seminal plasma or cellular debris) to viral transmission.

Although the proportion of ZIKV-positive sperm remained below 50%, the virus RNA persisted in sperm for up to 20 days post-infection. By 40 dpi, the viral positivity rate declined significantly, suggesting the onset of viral clearance or reduced replication activity. Interestingly, a subset of male hSTAT2 KI mice exhibited prolonged viral persistence, with ZIKV RNA still detectable in sperm at 40 and 60 dpi (Fig 6B). ZIKV can establish a persistent infection in the male reproductive tract of individual mouse, mirroring what has been observed in humans.

To assess the transmission kinetics over time, mating experiments were conducted using infected male mice at 5, 10, 20, and 40 dpi. In mated females, a progressive reduction was observed in both ZIKV RNA levels and the rate of viremia detection. The proportion of females with detectable viremia declined from 91.7% (after mating with males at 5 dpi in an independent experimental cohort) to 66.7% at 10 dpi, 33.3% at 20 dpi, and 0% at 40 dpi and 60 dpi (Fig 6D). Concordantly, the overall sexual transmission efficiency of male mice decreased from 100% at 5 dpi and 88.3% at 10 dpi to 50% by 20 dpi, with no transmission observed at 40 dpi and 60 dpi (Fig 6E). These results demonstrate a clear time-dependent decline in ZIKV sexual transmissibility, even though some viral RNA persisted in sperm.

**Fig 7:**
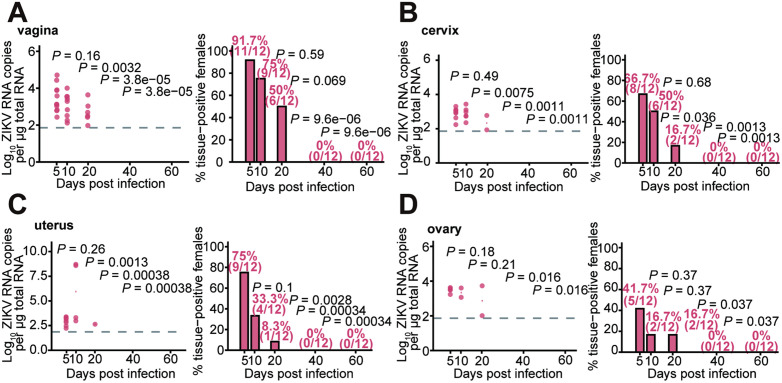
Viral loads and detection rates in the reproductive tract of females mated with males infected at various dpi. **(A)** Vagina. Left: viral RNA loads. Mann-Whitney U test vs. 5 dpi. Right: detection rates (n = 12 females). Fisher’s exact test vs. 5 dpi. **(B)** Cervix. Left: viral RNA loads. Mann-Whitney U test vs. 5 dpi. Right: detection rates (n = 12 females). Fisher’s exact test vs. 5 dpi. **(C)** Uterus. Left: viral RNA loads. Mann-Whitney U test vs. 5 dpi. Right: detection rates (n = 12 females). Fisher’s exact test vs. 5 dpi. **(D)** Ovary. Left: viral RNA loads. Mann-Whitney U test vs. 5 dpi. Right: detection rates (n = 12 females). Fisher’s exact test vs. 5 dpi.

Consistent with the decline in viremia, viral detection rates in the reproductive tissues of infected females also decreased over time. Positivity rates in the vagina, cervix, uterus, and ovaries showed a gradual downward trend (Fig 7A–7D).

Histopathological and immunohistochemical analyses were performed on females mated with males at 20 dpi. H&E staining revealed no significant histopathological abnormalities in the vagina, cervix, uterus, or ovary at either time point, with tissue architecture comparable to that observed at 5 dpi (S5A Fig). Immunohistochemical analysis further confirmed the presence of ZIKV E protein in the vaginal epithelium and scattered within the mucosal layer, with occasional positive signals detected in the cervical epithelium (S5B Fig). Viral antigen was also detected less frequently in the uterus and ovary at these later time points. Importantly, these findings collectively indicate that following sexual transmission from males infected for 20 days, viral antigen persists predominantly in the lower reproductive tract of females, with a distribution pattern similar to that observed during acute infection.

Collectively, these results demonstrate that the sexual transmissibility of ZIKV is strictly time-dependent. A high risk of transmission is confined to the early phase of infection (0–10 dpi), with the transmission window extending up to approximately 20 dpi. Beyond this period, the risk of sexual transmission declines sharply, and by 40 dpi, infected male hSTAT2 KI mice no longer exhibit sexual transmissibility, even some of them still had viral RNA in their semen. Therefore, the persistence of ZIKV RNA in sperm does not necessarily correspond to the actual duration of sexual transmission potential.

### Viral persistence and FcRn-mediated antibody transport in the male reproductive tract

**Fig 8:**
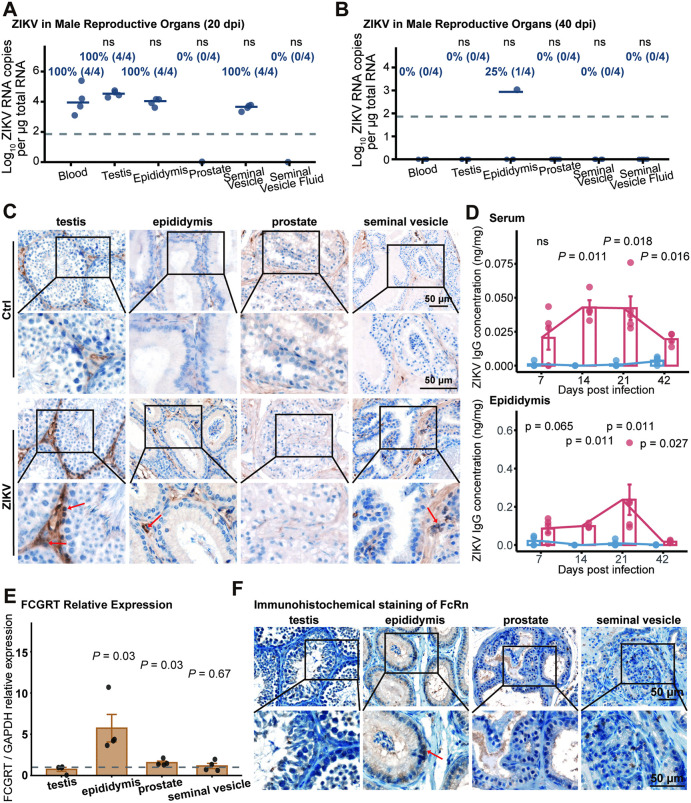
Viral persistence and FcRn-mediated antibody transport in the male reproductive tract. **(A)** Viral RNA loads in the blood, testes, epididymides, prostate, seminal vesicles, and seminal vesicle fluid detected by RT–qPCR at 20 dpi (n = 4). Statistical analysis was performed using the Mann-Whitney U test comparing each tissue to blood. **(B)** Viral RNA loads in the blood, testes, epididymides, prostate, seminal vesicles, and seminal vesicle fluid detected by RT–qPCR at 40 dpi (n = 4). Statistical analysis was performed using the Mann-Whitney U test comparing each tissue to blood. **(C)** Immunohistochemical staining for ZIKV E protein in male reproductive organs at 20 dpi. Representative images of testes, epididymides, prostate, and seminal vesicles are shown. Positive signals (brown) indicate ZIKV E protein. Sections were counterstained with hematoxylin. Red arrows indicate representative positive signals. (Scale bar, 50 μm). **(D)** ELISA quantification of ZIKV-specific IgG antibodies in serum and epididymal homogenates. Samples were collected at 7, 14, 21, and 42 dpi (n = 4 per time point). Data are presented as mean ± SEM. Statistical analysis was performed using the Mann-Whitney U test comparing epididymal homogenates to serum at each time point. **(E)** Relative Fcgrt mRNA expression in different male reproductive organs from control male mice, normalized to *Gapdh* using the 2^–ΔΔCt method. Each data point represents an individual mouse (n = 4 per group). Horizontal bars indicate mean ± SEM. Statistical significance was determined by Wilcoxon rank-sum test comparing epididymis versus testis. **(F)** Immunohistochemical staining for FcRn in male reproductive tissues from control male mice. Representative images of testes, epididymides, prostate, and seminal vesicles are shown. Positive signals (brown) indicate FcRn expression. Red arrows indicate positive signals. (Scale bar, 50 μm).

To elucidate the mechanism of the decline in sexual transmissibility—from 100% at 5 dpi to 50% at 20 dpi—in hSTAT2 KI male mice, we examined their reproductive tracts at 20 dpi. Consistent with our previous findings [20], hSTAT2 KI mice are capable of long-term ZIKV carriage. ZIKV RNA remained detectable in the blood, testes, epididymides, and seminal vesicles at 20 dpi (Fig 8A), whereas no viral RNA was detected in the prostate or seminal vesicle fluid. At 40 dpi, viral RNA was only detected in the epididymis of one out of four mice, with no detectable virus in the blood or other reproductive tissues (Fig 8B). This finding suggests that long-term viral persistence in the epididymis may be associated with chronic shedding of ZIKV in sperm, potentially contributing to the sustained but low-level sexual transmission risk at later time points.

Immunohistochemical analysis confirmed ZIKV E protein in the male reproductive system at 20 dpi (Fig 8C). In the testis, ZIKV-positive signals were predominantly in the interstitium, with candidate cell types including Leydig cells, macrophages, and other infiltrating immune cells, and occasional positive signals within the seminiferous tubules (Sertoli or spermatogenic cells). In the epididymis, viral antigen was detected in the interstitium and occasionally in epithelial cells lining the duct. In the seminal vesicle, positive staining was observed in the interstitium and scattered within mucosal folds. No specific ZIKV staining was detected in the prostate, consistent with the absence of viral RNA. The distribution pattern suggests viral persistence and clearance differ among male reproductive glands. The absence of viral RNA in the prostate and seminal vesicle fluid likely contributes to reduced sexual transmission efficiency at 20 dpi, whereas residual virus in the testes, epididymides, and seminal vesicles may sustain low-level infectious potential. Thus, temporal kinetics of ZIKV sexual transmissibility reflect a balance between prolonged viral shedding and clearance across distinct reproductive organs.

Interestingly, the observed decline in sexual transmission coincided with the onset of host antibody production. To explore this correlation, we measured ZIKV-specific IgG levels in the serum and epididymal homogenates of hSTAT2 KI male mice at 7, 14, 21, and 42 dpi, together with samples from uninfected control mice. Compared with controls, serum antibody titers increased markedly at 14, 21 dpi and 42 dpi, and a similar significant increase was detected in epididymal homogenates at the same time points (Fig 8D), indicating local presence of antiviral antibodies within the reproductive tract. The neonatal Fc receptor (FcRn) is well known to mediate IgG transcytosis across epithelial barriers and contributes to IgG homeostasis and adaptive immunity [21]. RT–qPCR analysis revealed that *Fcgrt*, the gene encoding FcRn, was expressed at substantially higher levels in the epididymis than other male reproductive tissues, with expression levels normalized to the housekeeping gene *Gapdh* (Fig 8E). Consistent with this, immunohistochemical staining demonstrated prominent FcRn localization within the epithelial cells of the epididymis, with negligible staining observed in other reproductive organs (Fig 8F).

The timing of the antibody accumulation coincided with the decline of sexual transmissibility, suggesting that FcRn-mediated IgG transport into the epididymis may neutralize viral particles and thereby restrict viral shedding.

Collectively, these results suggest that the decreased sexual transmission efficiency of ZIKV during prolonged infection is associated with two major mechanisms: the clearance of infectious virus from seminal vesicle fluid, which reduces the viral load available for transmission; and FcRn-mediated accumulation of antiviral IgG antibodies in the epididymis, which may neutralize viral particles and limit their dissemination via semen. Together, these processes likely contribute to the time-dependent decline in ZIKV sexual transmissibility observed in hSTAT2 KI mice.

## Discussion

In this study, we established a ZIKV sexual transmission model using hSTAT2 KI mice. The kinetics of sexual transmission were systematically evaluated across different time points and viral strains. In addition, infection stage–dependent male factors influencing transmission, as well as the effects on the reproductive tract of nonpregnant females, were further evaluated.

Sexual behavior involves both the intended and unintended exchange of multiple body fluids across various anatomical sites. Studies using immunodeficient mice have demonstrated that intravaginal inoculation shows milder clinical manifestations and a more localized ZIKV infection compared with natural sexual transmission [22]. In contrast, our model detected viral RNA in multiple female reproductive tissues, including the vagina, cervix, uterus, and ovaries. Owing to the intrinsic differences between sexually transmitted infection and intravaginal inoculation, this model provides distinct advantages and substantial scientific value for accurately reproducing the sexual transmission process of Zika virus in humans.

Importantly, the disease manifestations in hSTAT2 KI mice differ markedly from those observed in highly immunodeficient models such as *Ifnar1*^−/−^ or AG129 mice, which typically develop severe disease, including weight loss and mortality, following ZIKV infection [23,24]. In contrast, ZIKV infection in hSTAT2 KI mice resulted in mild, self-limited clinical manifestations, with no mortality and only transient body weight changes [16]. This attenuated disease course more closely resembles the asymptomatic or mild infections commonly seen in humans, thereby enabling the investigation of long-term viral persistence and sexual transmission dynamics. Indeed, our previous work demonstrated prolonged ZIKV RNA persistence in the testes of hSTAT2 KI mice, supporting the utility of this model for studying chronic infection in the male reproductive tract [20].

During the ZIKV outbreak in Barranquilla, Colombia, mathematical modeling estimated that approximately 23% of infections were attributable to sexual transmission [25]. Clinical reports further indicated a median interval of about 13 days between symptom onset in primary and sexually transmitted cases [26]. These epidemiological data suggest that ZIKV sexual transmission frequently occurs during the early viremic phase and at a notably high rate, although its detection is often masked by the dominant mosquito-borne route. This frequency of sexual transmission is consistent with our observations in hSTAT2 KI male mice, which exhibited high sexual transmission efficiency during early infection (0–10 dpi), with the transmission window extending up to 20 dpi. Notably, most reported human cases of sexual transmission occurred similarly within 19 days after the onset of signs in the man [5]. Collectively, the hSTAT2 KI mouse model provides an experimental platform for investigating the kinetics and mechanisms of ZIKV sexual transmission, as well as for evaluating potential intervention strategies.

Remarkably, bilateral epididymal ligation in male mice during early infection did not abolish sexual transmission, as half of mated females still became infected. This observation, consistent with previous findings in immunodeficient mouse models [27] and clinical reports of vasectomized men transmitting ZIKV [28], indicates that sperm are not the sole vehicle of sexual transmission.

Comparative analysis of viral strains revealed that the African and Asian lineages differ by more than 50 amino acids in the nonstructural proteins NS1, NS2B, and NS5 [29], differences that confer greater neurovirulence and lethality in the African lineage [30]. Although sexual transmission of ZIKV was not conclusively documented until the outbreak in Latin America in 2016 [31], a returning traveler from Senegal was suspected of sexually transmitting the virus to his wife as early as 2008 [32]. Due to limited epidemiological surveillance before 2016, the sexual transmissibility of early African strains was likely underrecognized. Our results demonstrate that sexual transmissibility is an evolutionarily conserved feature of ZIKV, already present in the ancestral African lineage. This conclusion is supported by prior findings of persistent viral replication in the male reproductive tract and semen [33], as well as vaginal infection in nonhuman primates [34]. Collectively, these findings suggest that sexually transmitted ZIKV infections may have occurred long before their first clinical recognition, highlighting the importance of incorporating sexual transmission into future outbreak surveillance, risk assessment, and public health strategies.

Mechanistically, our study demonstrates that ZIKV sexual transmission involves more contributing factors than previously recognized. Although the prolonged presence of ZIKV in the testes and sperm is generally been considered the primary determinant of sexual transmission [35,36], non-sperm components of semen also appear to play a substantial role. Epididymal ligation blocks the release of spermatozoa and epididymal-derived contents but does not eliminate the majority of ejaculatory fluid. Seminal plasma is produced mainly by the accessory sex glands, with approximately 65–75% originating from the seminal vesicles and ~30% from the prostate, whereas testicular and epididymal secretions contribute only a minor proportion (2–5%) of the total ejaculate [37]. Thus, even in the absence of sperm transport, free virions and infected cellular components derived from accessory glands may remain available for transmission. Consistent with this possibility, we detected ZIKV E protein in EpCAM⁺ epithelial cells and F4/80⁺ macrophages within seminal vesicle fluid, suggesting that non-sperm cellular components of the ejaculate may serve as vehicles for viral dissemination. Previous studies have similarly reported the presence of ZIKV in non-sperm cells isolated from semen of infected patients [38]. These cells, originating from the male reproductive tract, may serve as critical contributors to viral transmission, supported by evidence of persistent ZIKV infection across multiple male reproductive organs in nonhuman primates [39]. While the testes constitute an immune-privileged niche that facilitates long-term viral persistence, infection of accessory glands such as the epididymis, prostate, and seminal vesicles may also sustain sexual transmission, analogous to mechanisms observed in HIV [40]. Our findings reveal that ZIKV persists for extended periods in the testes, epididymides, and seminal vesicles, suggesting that these organs act as potential viral reservoirs contributing to prolonged infectious potential.

Although ZIKV RNA remains detectable in semen up to 60 dpi, sexual transmission efficiency declines markedly by 20 dpi. This reduction likely reflects combined effects of infectious virus clearance and the emergence of host antibodies. The FcRn, which mediates IgG transport across epithelial barriers, plays a pivotal role in mucosal immune defense [21]. Consistent with previous reports [41], we observed relatively high FcRn expression in the epididymal epithelium and detected increasing levels of ZIKV-specific IgG within epididymal homogenates during the period when sexual transmission efficiency declined. These observations raise the possibility that FcRn-mediated IgG transport may contribute to the local accumulation of antiviral antibodies within the male reproductive tract. Such antibodies could potentially neutralize infectious viral particles and reduce viral shedding into semen, even when viral RNA remains detectable. Although the mechanisms remain to be elucidated, we propose that efficient clearance of infectious virus from semen, together with FcRn-mediated antibody transport, collectively contributes to the observed decline in sexual transmissibility during ZIKV infection. Future studies using FcRn blockade, genetic disruption, or direct measurements of neutralizing activity in reproductive tract secretions will be required to determine whether FcRn-dependent antibody transport contributes to the decline in ZIKV sexual transmissibility.

Several technical limitations of this study should be acknowledged. First, tissues were not perfused prior to collection, so residual blood may have contributed to viral RNA signals. However, tissue-specific distribution patterns of viral antigen differed markedly, and immunohistochemistry localized viral antigen within specific anatomical compartments, confirming tissue-associated virus independent of blood. Nevertheless, a minor contribution from residual blood cannot be fully excluded. Second, tissue viral loads were not measured from the same males used in mating; instead, a parallel cohort was used, introducing potential inter‑individual variability.

The consequences of ZIKV sexual transmission on the reproductive health of nonpregnant females remain uncertain [42]. In the present study, we observed that although ZIKV RNA could be detected in multiple female reproductive organs following sexual transmission, the most pronounced transcriptional alterations at the acute time point examined were confined to the vagina, with minimal changes detected in the upper reproductive tract. Specifically, we noted a downregulation of genes associated with lipid metabolism in vaginal tissue, a pattern that differs from the interferon-dominated responses reported in intravaginal inoculation models [15,17]. Whether this localized metabolic reprogramming reflects alterations in seminal composition following male infection [43], or whether it has any bearing on longer-term reproductive outcomes, remains to be determined. It is important to emphasize that our analysis was restricted to an early acute phase (one day post-mating), and later dissemination to upper reproductive tract tissues cannot be excluded. Notably, preliminary observations from ongoing studies in parallel cohorts suggest that offspring derived from sexually infected females may exhibit altered developmental parameters; however, these findings are beyond the scope of the current report and will require dedicated investigation in future work. Thus, while acute pathological changes in nonpregnant females appear limited, the potential for delayed or downstream reproductive consequences warrants further examination.

In summary, we demonstrated that sexual transmission of ZIKV occurs within a finite window, which is shorter than the prolonged viral RNA shedding in semen. This process involves both sperm and non-sperm components, with multiple male reproductive organs serving as substantive viral reservoirs. The subsequent decline in transmissibility may be attributed to mechanisms such as FcRn-mediated antibody transport. In female mice, infection remains predominantly localized to the vaginal epithelium. Together, this model provides key insights into the dynamic balance between viral persistence and host-mediated clearance.

## Methods

### Ethics statement

All animal experiments were approved by the Animal Ethics Committee of Capital Medical University (AEEI-2024–140) and conducted in accordance with institutional and national guidelines for animal welfare.

### Animals

Male and female human STAT2 knock-in (hSTAT2 KI) mice (C57BL/6J background, 6–8 weeks old) were purchased from the Jackson Laboratory. All mice were housed under specific pathogen-free (SPF) conditions at 20–26°C and 40–70% humidity, with a 12 h light/dark cycle and free access to food and water.

### Virus and infection

ZIKV Asian strain SMGC-1 (GenBank accession no. KX266255) was originally isolated in Shenzhen, China, from an imported ZIKV patient in 2016. ZIKV African strain MR766 (GenBank accession no. ON123672) was kindly provided by Dr. Gong Cheng (Tsinghua University). Both virus strains were propagated in *Aedes albopictus* C6/36 cells (National Infrastructure of Cell Line Resource, Cat#3131C0001000400021). Viral titers were determined by plaque assay on Vero cell monolayers under overlay medium containing 1.1% methylcellulose. Male hSTAT2 KI mice were intraperitoneally inoculated with 1 × 10⁴ PFU of ZIKV in PBS; control mice received an equal volume of PBS.

### Establishment of the sexual transmission model

ZIKV-infected male mice were co-housed with uninfected female hSTAT2 KI mice at a 1:2 male-to-female ratio for 3 days at 5, 10, 20, and 40 days dpi. One day after co-housing, female blood and reproductive tissues (vagina, cervix, uterus, and ovaries) were collected for viral load quantification and histopathological analysis.

### Sample collection

Blood samples were obtained via retro-orbital plexus puncture at necropsy. Tissue samples were divided for RNA extraction and 4% paraformaldehyde fixation.

Epididymal spermatozoa were isolated at necropsy following established protocols [44]; briefly, male mice, were euthanized by cervical dislocation, both caudae epididymides and vas deferentia were dissected, minced in pre-warmed medium, and incubated to allow sperm release. Part of the sample was used for sperm smears, and the remainder was centrifuged at 1000 rpm for RNA extraction.

Seminal vesicle fluid was also collected for smear preparation and viral detection.

### Viral RNA quantification and gene expression analysis

Total RNA was extracted from blood and tissue samples using TRIzol reagent (Transgen China, ET101–01) according to the manufacturer’s protocol. ZIKV RNA was quantified by one-step quantitative RT–PCR (Quant One Step qRT-PCR Kit, Tiangen, China) on a 7500 Real-Time PCR System (Applied Biosystems, USA) as previously described [36]. Viral RNA copies were determined using the standard curve method. Briefly, ZIKV genomic RNA was transcribed in vitro, quantified, and serially diluted to generate a standard curve ranging from 7.325 × 10 to 7.325 × 10^5^ copies per reaction. The standard curve showed excellent linearity: y = –0.2967x + 10.095, R² = 0.998. The lower limit of detection (LOD) was 73.25 copies per reaction, defined as the lowest concentration yielding a detectable signal in ≥95% of replicate assays. Samples with Ct values exceeding this threshold were considered negative. Viral RNA levels were expressed as copies per μg of total RNA extracted from each sample.

For relative quantification of Fcgrt mRNA, expression levels were normalized to Gapdh and calculated using the 2^–ΔΔCt method. Primer sequences were as follows:

ZIKV forward: 5′-TCAGACTGCGACAGTTCGAGT-3′;
ZIKV reverse: 5′-GCATATTGACAATCCGGAAT-3′;
*Gapdh* forward: 5′-AATGTGTCCGTCGTGGATCTGA-3′;
*Gapdh* reverse: 5′-GATGCCTGCTTCACCACCTTCT-3′;
*Fcgrt* forward: 5′- AACCCATCTACGGGGCTTC-3′;
*Fcgrt* reverse: 5′- GTCCCCCTCTTCTGACCATTTA-3′

To further validate our results, a subset of samples was analyzed using a probe-based RT-qPCR assay as described previously [45], which confirmed the viral RNA detection patterns obtained with the SYBR Green method.

### Hematoxylin and Eosin (H&E) staining

Tissues were fixed in 4% paraformaldehyde, paraffin-embedded, and sectioned at 5 μm thickness. Sections were deparaffinized, hydrated, stained with hematoxylin for 12 min and eosin for 20 min, dehydrated through an alcohol gradient, cleared with xylene, and mounted with neutral resin. Images were acquired using a Grundium Ocus portable digital microscope slide scanner (Grundium Oy, Tampere, Finland). Only uniform adjustments to brightness and contrast were applied equally across entire images; no selective alterations or region-specific manipulations were performed.

### Immunohistochemistry (IHC)

Immunohistochemical staining was performed as described previously [46]. Briefly, paraffin sections were deparaffinized, rehydrated, and permeabilized with 0.3% Triton X-100, followed by heat-induced antigen retrieval in citrate buffer (pH 6.0). Sections were incubated with peroxidase blocking reagent and 1% BSA, then with primary antibodies overnight at 4°C. For ZIKV E protein detection, mouse anti-ZIKV E monoclonal antibody 4G2 (1:500) was used. For FcRn detection, rabbit anti-Fcgrt polyclonal antibody (PA5–97111, Invitrogen, 1:200) was used. After primary antibody incubation, sections were incubated with HRP-conjugated secondary antibodies for 20 min at 37°C using the UltraSensitive SP kit (PV-9000 for mouse primary, PV-9001 for rabbit primary; ZSGB-BIO, Beijing, China) following the manufacturer’s instructions. Signal was developed with DAB substrate, and slides were examined using a Grundium Ocus portable digital microscope slide scanner (Grundium Oy, Tampere, Finland) [43] to assess antigen localization and distribution.

### Immunofluorescence staining

Immunofluorescence staining was performed as previously described [13]. Semen smears were fixed, permeabilized, and blocked before incubation with primary antibodies at 4 °C overnight. After washing with PBS, slides were incubated with fluorescent secondary antibodies for 1 h in the dark and counterstained with DAPI (B1061, PPL). Fluorescence was observed using an Olympus IX71 microscope.

Primary antibodies: mouse anti-ZIKV E (4G2, 1:500), rabbit anti-F4/80 (1:100, Abcam, ab111101), rabbit anti-DDX4 (1:100, Abcam, ab13840), and mouse anti-EpCAM (1:200, Invitrogen, MA5–12436).

Secondary antibodies: donkey anti-mouse IgG (Alexa Fluor 488, A21202, 1:1000) and donkey anti-rabbit IgG (Alexa Fluor 594, A21207, 1:1000).

### Papanicolaou staining

To assess sperm morphology alongside viral antigen detection, sperm smears were subjected to Papanicolaou staining following immunohistochemical development. After DAB staining for ZIKV E protein, the same smears were processed using a commercial Papanicolaou staining kit (BA-4033A, BA-4035A, Baso Diagnostics Inc.) per the manufacturer’s instructions. Briefly, slides were fixed in 95% ethanol, hydrated through graded ethanol, and stained sequentially with hematoxylin, Orange G6, and EA50, with dehydration and clearing steps as specified.

Papanicolaou staining differentially colors sperm components: nuclei appear blue-purple (hematoxylin), acrosomes orange-pink (Orange G6), and cytoplasmic/tail structures blue-green (EA50), enabling detailed morphological evaluation [47]. This staining is used exclusively for cytomorphological assessment and does not detect viral antigen.

### Transcriptomic analysis

Vaginal, cervical, uterine, and ovarian tissues from control and ZIKV-infected female mice (n = 3) were subjected to bulk RNA sequencing, performed by Majorbio (Shanghai, China). Library preparation utilized the Illumina NovaSeq platform with paired-end 150 bp reads.

Raw sequencing data have been deposited in the National Genomics Data Center (CNCB) under accession number PRJCA059116 (https://ngdc.cncb.ac.cn). Data visualization was completed in R (v4.3.2) using custom scripts. All custom R scripts used for data analysis and figure generation are provided as Supplementary S1 File to ensure reproducibility.

### Epididymal ligation surgery

Bilateral epididymal ligation was performed under anesthesia as described previously [48], Sham-operated males underwent exposure of the epididymis without ligation.

### Antibody detection

ZIKV-specific IgG levels in serum and epididymal homogenates were measured by ELISA (SPS-22995, SenBeiSen Biotech).

### Plaque assay

Vero cells were seeded in 12-well plates and grown to 90–100% confluence. Blood samples or tissue homogenates were centrifuged at 12,000 × g for 10 min at 4°Cto remove debris. The supernatant (200 μL per well) was added to Vero cell monolayers and incubated at 37°C for 1 h with gentle rocking every 15 min to allow viral adsorption. After adsorption, the inoculum was removed, and cells were overlaid with 1.1% methylcellulose in MEM supplemented with 2% fetal bovine serum. Plates were incubated at 37°C in 5% CO₂ for 7 days. The overlay was then removed, and cell monolayers were fixed and stained with 0.5% crystal violet in 20% methanol for 30 min at room temperature. Plaques were counted visually, and viral titers were calculated as plaque-forming units (PFU) per mL for blood samples or PFU per mg tissue for tissue samples.

### Statistical analysis

Data are presented as individual data points with median (horizontal bar) for viral RNA loads, as mean ± SEM for body weight and gene expression, or as percentages for detection and transmission rates. Statistical analyses were performed using R software (version 4.3.2). For comparisons of viral RNA loads between two independent groups (e.g., ZIKV vs. Ctrl, or different time points vs. 5 dpi), the Mann-Whitney U test (Wilcoxon rank-sum test) was applied due to the small sample sizes and non-normal distribution of the data. For comparisons of infection rates, detection rates, pregnancy rates, and transmission rates, Fisher’s exact test was used. Body weight changes over time were analyzed using the Mann-Whitney U test at each time point. For comparisons involving more than two groups (e.g., multiple tissues), pairwise Mann-Whitney U tests were performed, and p-values were adjusted using the Bonferroni method where indicated. All graphical visualizations were generated using the ggplot2 package (version 3.4.4).

## Supporting information

S1 Fig Physiological parameters and viremia in females mated with ZIKV-infected males at 5 dpi. (A) Plaque assay of blood and vaginal homogenate supernatants from co-housed females. Blood and vaginal samples were collected from female mice one day after mating with ZIKV-infected or control males at 5 dpi. Homogenate supernatants were incubated with Vero cell monolayers, and plaques were visualized by crystal violet staining. Statistical significance was determined using a t-test, with *P* values indicated in the figure. (B) Average body weight of females co-housed with ZIKV-infected or control males were measured at 5 dpi. Data are presented as mean ± SEM (n = 3 per group). Statistical analysis was performed using the Mann-Whitney U test. (C) Viral RNA loads in blood. Blood samples were collected from female mice one day after mating with ZIKV-infected or control males at 5 dpi. Statistical analysis was performed using the Wilcoxon rank-sum test.
(TIF)

S2 Fig Transcriptional profiling of female reproductive tissues following sexual transmission of ZIKV. (A) Expression of lipid metabolism-related genes. (B) Expression of cell death-related genes. (C) Expression of interferon response-related genes. Data are presented as mean ± SEM (n = 3 per group). Statistical analysis was performed using the Mann-Whitney U test.
(TIF)

S3 Fig Clinical course and histopathology in reproductive organs of ZIKV-infected male hSTAT2 KI mice. (A) Body weight changes of male hSTAT2 KI mice (n = 4). Data are presented as mean ± SEM. Statistical analysis was performed using the Mann-Whitney U test. *p < 0.05, **p < 0.01. (B) Survival rates of mice post-inoculation (n = 4). Data are presented as Kaplan-Meier survival curves. (C) Representative H&E-stained sections of the male reproductive tract (testes, epididymides, prostate, and seminal vesicles were collected at 5 dpi). (Scale bar, 50 μm). (D) Plaque assay of blood and seminal vesicle fluid homogenate supernatants from males. Blood and seminal vesicle fluid samples were collected from male mice at 5 dpi. Homogenate supernatants were incubated with Vero cell monolayers, and plaques were visualized by crystal violet staining. Statistical significance was determined using a t-test, with *P* values indicated in the figure.
(TIF)

S4 Fig Immunohistochemical staining of female cervix, uterus, and ovary following ZIKV infection. Left: Asian strain (SMGC-1). Right: African strain (MR766). (A) Cervix. (B) Uterus. (C) Ovary. Scale bar, 50 μm.
(TIF)

S5 Fig Histopathology and viral distribution in females infected sexually from 20-day-infected males. (A) H&E staining of female reproductive tissues. (B) Immunohistochemical staining of ZIKV E protein in the reproductive tract. (Scale bar, 50 μm), positive signals indicated by red arrows.
(TIF)
